# The Poor Man's Home.—XIII

**Published:** 1897-11-13

**Authors:** 


### Modern Sociology.

### THE POOR MAN'S HOME.—XIII.

### Master and Man—(*concluded*).

The examples of housing by employers which we are now about to describe are not carried out on strict commercial principles. It must therefore be remembered that, while they show how much an employer of high ideals may do for the well-being of his workers, it does not imply a duty which all employers ought to fulfil. In many businesses it is impossible for the master to house his workmen; in others the workmen themselves would not be grateful for such provision, preferring to choose dwellings for themselves. This being conceded, it still remains a fact that such firms as Messrs. Lever and Mr. Cadbury confer benefits on their workers which make it a desirable thing to be numbered among their employés, and that while they themselves reap no direct commercial benefit from the provision of good houses, they are certain by doing so to obtain the services of a better class of workers, who will, moreover, take more care than usual to avoid doing anything which will involve the loss of their comfortable homes. We can conceive of a strike being prevented by the thought that it implies leaving a good house.

Messrs. Lever have, at Port Sunlight, created a village which is not only healthy but picturesque. Port Sunlight looks like an old English town, with certain variations. The chief of these are that the streets are wide and shaded with trees, that the quaint cottages are commodious, that the sanitary arrangements are good, that the houses are provided with gas and water (both hot and cold), and that there is a well-paved yard at the back, and a garden in front. Two of the houses are exact copies, so far as external appearance goes, of Shakespeare's birthplace. The men's club and the village shop are built in similar picturesque fashion; and the village schools, though of a modern type, are not out of harmony with the surrounding houses. The rents are very low—for, indeed, only enough is charged to allow for repairs and depreciation, and interest on money expended—for Messrs. Lever regard the cottages as part of the expenses of the business. The business as a whole returns a profit, and the masters regard this housing scheme as a form of profit-sharing, in which the wives and children of their workers may participate. The widows of workmen are, moreover, permitted, at the discretion of the firm, to continue occupying the homes they had in their husbands' lifetime. Lodgers who are employés of the firm may be taken, but under strict regulations. Only childless couples, or those who have not more than two children, who must be under twelve years of age, are allowed to take in lodgers, and these must always be of the same sex. The number that can be taken is also carefully regulated according to the size of the house. The average cottage contains a large kitchen and scullery, three bedrooms, and a bathroom. The rent is 3s. a week. The kitchen measures 15 ft. 3 in. by 18 ft.; the space behind it, 9 ft. in depth, being divided into scullery, pantry, and bathroom. On the upper floor are three bedrooms, the largest measuring 15 ft. 3 in. by 15 ft., another 12 ft. by 10 ft., and the third 9 ft. by 8 ft. For 5s. a week a house with a parlour and an extra bedroom may be had. There are no taxes, not even a water-rate. Gardens can be rented, eight rods of ground for 1s. a year, and water-pipes are led into them also. Port Sunlight also possesses a club and bowling-green, a girl's institute, classes for technical education, a reading-room, a billiard-room, a lecture hall, a village council—in short, all the requisites for an intelligent civic life. The firm seems satisfied that the results of their venture will prove wise as well as beneficent.

Another beautiful village is being created at Bournville, near Birmingham, by Mr. George Cadbury. Mr. Cadbury does not, however, wish to keep the houses in his own hands. His object is rather to induce working men to become their own landlords. To this end he is prepared to advance money to people who desire to own their homes at very low rates. To secure a desirable class of residents, however, some safeguards are necessary. Thus, none of the houses are to cost less than £150, which will of itself exclude the lowest classes. Moreover, anyone who wishes to profit by Mr. Cadbury's generosity must be able to put down at once some portion of the cost of his house. If he can give half, the remainder will be advanced to him at 2½ per cent. interest. If he can pay only a smaller proportion, the rate of interest is 3 per cent. In fact, the capacity for saving which a man shows by having a part of the purchase money in hand is the best guarantee for the repayment of the remainder. And at this rate he lives at a very cheap rent. Mr. Cadbury reckons thus: Suppose a house costs £150, and £30 is paid towards it. The ground rent and interest should be more than covered by the produce of the large garden, especially if poultry should be kept. The interest on £120 at 3 per cent. is 72s. per annum, or 1s. 4½d. per week. This is each year reduced by about 1½d. per week, if £10 a year is paid towards the principal advanced. That is, the tenant pays a rent of 5s. 2¾d. a week, and at the end of about thirteen years the house is his own. The ordinary rent for a house of this class is 6s. a week, and the tenant never becomes the owner of it. Taking as our example the man who has paid £30 in advance and borrowed the rest, the sum works out thus:—

| | | | |
|---|---|---|---|
| Payment in advance ... ... ... ... ... | £30 | 0 | 0 |
| Twelve yearly payments ... ... ... ... | 120 | 0 | 0 |
| Interest diminishing yearly for 12 years ... | 23 | 8 | 0 |
| Total payments ... ... ... ... | £173 | 8 | 0 |

Had he rented the house at 6s. a week, he would at the end of 12 years have paid £187 4s., and not a brick of it would be his own.

In the event of a tenant dying before he has paid up for his house, Mr. Cadbury is willing to buy it at cost price. If the development of the estate warrants it, Mr. Cadbury proposes to give a piece of land of about seven acres for a recreation-ground for the tenants, as well as land for schools, baths, and an institute, to be managed by a committee of the tenants.

The dwellings erected by Messrs. James Smieton and Sons, jute manufacturers, of Carnoustie, near Dundee, are of older date, but are comfortable, substantial houses. They consist of a kitchen which measures 15 ft. by 12 ft., a parlour of the same size, and a bedroom 12 ft. by 8 ft. The rent of such a house is £7 11s. 8d. Tenants are not allowed to purchase their dwellings, as they are intended solely for the employés of the firm. Rents are deducted from wages, so that there is no loss from this source. As at Port Sunlight, the housing accounts are not kept separate from the general accounts of the company; but the proprietors estimate that the houses give them a profit of 3 per cent. Yet the rent of a similar lodging in the neighbourhood would be £10 10s.

